# Seasonal and inter-annual variation in the chlorophyll content of three co-existing *Sphagnum* species exceeds the effect of solar UV reduction in a subarctic peatland

**DOI:** 10.1186/s40064-015-1253-7

**Published:** 2015-09-04

**Authors:** Anna Hyyryläinen, Pasi Rautio, Minna Turunen, Satu Huttunen

**Affiliations:** Department of Biology, University of Oulu, P.O. Box 3000, 90014 Oulu, Finland; The Natural Resources Institute Finland, P.O. Box 16, 96300 Rovaniemi, Finland; Arctic Centre, University of Lapland, P.O. Box 122, 96101 Rovaniemi, Finland

**Keywords:** *Sphagnum balticum*, *Sphagnum jensenii*, *Sphagnum lindbergii*, Chlorophyll, UVB, Peatlands

## Abstract

We measured chlorophyll (chl) concentration and chl *a/b* ratio in *Sphagnum balticum*, *S. jensenii*, and *S. lindbergii*, sampled after 7 and 8 years of ultraviolet-B (UVB) and temperature manipulation in an open field experiment in Finnish Lapland (68°N). We used plastic filters with different transmittance of UVB radiation to manipulate the environmental conditions. The plants were exposed to (1) attenuated UVB and increased temperature, (2) ambient UVB and increased temperature and (3) ambient conditions. Chlorophyll was extracted from the capitula of the mosses and the content and *a/b* ratio were measured spectrophotometrically. Seasonal variation of chlorophyll concentration in the mosses was species specific. Temperature increase to 0.5–1 °C and/or attenuation of solar UVB radiation to ca. one fifth of the ambient (on average 12 vs. 59 uW/cm^2^) had little effect on the chlorophyll concentration or its seasonal variation. In the dominant *S. lindbergii*, UVB attenuation under increased temperature led to a transient decrease in chlorophyll concentration. Altogether, species-specific patterns of seasonal chlorophyll variation in the studied *Sphagna* were more pronounced than temperature and UVB treatment effects.

**Electronic supplementary material:**

The online version of this article (doi:10.1186/s40064-015-1253-7) contains supplementary material, which is available to authorized users.

## Background

*Sphagna* are crucial in forming and sustaining peatlands—specific ecosystems that maintain a unique diversity of habitats and species (Clymo and Hayward 1982; Vitt 2000; Rydin et al. 2006; Rydin and Jeglum 2006) and function as a large store of organic carbon, particularly in boreal and subarctic areas (Gorham 1991; Limpens et al. 2008). As a key component of the ecosystem, they affect many processes within it; therefore, the changes triggered in *Sphagna* by a changing environment may be reflected in the entire ecosystem. This is important, as they are also susceptible to changes in the environment, including shifts in UVB and temperature regime (e.g. Dorrepaal et al. 2003; Huttunen et al. 2005; Wiedermann et al. 2007; Gignac 2011), principally due to their specific structure (leaves of one cell layer, absence of waxes or any other protective surface structures). In open peatlands, *Sphagnum* mosses are not shielded by tree canopies and can be exposed to high levels of UVB irradiance. Given the importance of *Sphagna* for the sustainability of peatlands, considering recent increased solar UVB irradiance (Weatherhead et al. 2005) and elevated temperatures at high latitudes (Jylhä et al. 2004; McBean 2005), it is essential that we understand how both these factors affect peat mosses.

Solar UV radiation is an important environmental factor, regulating growth, biochemistry and some structural features in bryophytes (Gehrke 1998; Rozema et al. 2005; Caldwell et al. 2007). UV effects on mosses are species-specific, possibly reflecting differences in desiccation tolerance (Csintalan et al. 2001). *Sphagnum* mosses growing under near-ambient conditions seem to be able to hold more water as they are shorter and stouter, and have larger capitula, than those growing under attenuated UVB (Robson et al. 2003, 2004). Changes in the intensity of different wavelengths of solar irradiance affect shape and size of chloroplasts (Glime 2007), as well as pigment concentration in bryophytes (Niemi et al. 2002a, b).

Even a small enhancement (by less than 1 °C) of summer temperatures may have a significant effect on growth and cover density of peat mosses (Dorrepaal et al. 2003). These responses are species-specific, and seem to correlate with the requirements of a particular species to environmental conditions (Gunnarsson et al. 2004; Breeuwer et al. 2008; Haraguchi and Yamada 2011). Differences in responses to increased temperatures may, in the long run, lead to changes in species composition in a given ecosystem. Where increased temperatures do not directly affect peatmosses, they may still have an impact on vascular plants (Weltzin et al. 2000). As a consequence, species range and ratio may change, and that may be hazardous for the ecosystem.

The photosynthetic pigments in *Sphagna* are chlorophyll *a* and *b*, and carotenoids. The absorption spectra of the two chlorophylls (chls) differ slightly (Porra et al. 1989), and plants use these differences to adapt to varying light conditions. In mosses, the total chl content and *a/b* ratio changes depending on factors such as light availability, plant vitality, growth phase, and various environmental stresses (Baxter et al. 1992; Martínez-Abaigar et al. 1994; Gerdol 1996; Martínez-Abaigar and Núñiez-Olivera 1998; Bonnett et al. 2010). A deficit of light often promotes chl *b* production, reducing the chl *a/b* ratio. Total chl content is generally higher in species that grow in shady habitats, and lower in those commonly found in open sunny sites (Martin and Churchill 1982; Martínez-Abaigar and Núñiez-Olivera 1998). During the growing season, the chl content normally increases with decreasing light intensity. Seasonal pigment variation is more pronounced in species growing in varying light conditions, than those growing in permanently shady or sunny habitats (Kershaw and Webber 1986; Martínez-Abaigar and Núñiez-Olivera 1998). In *Sphagna*, chl content is species-specific (Gehrke 1998; Niemi et al. 2002a, b) and it depends on the ecological requirements of the species (Yefremov et al. 2000; Naumov and Kosykh 2011). Night chilling may cause rapid degradation of chls in some *Sphagna* (Gerdol et al. 1994).

In *Sphagnum* mosses, a positive correlation between chl concentration and net photosynthesis has been recorded (Gaberščik and Martinčič 1987). Thus, environmental factors affecting the chl content in peat mosses may also affect the rate of photosynthesis. Bryophytes from open sunny habitats are normally adapted to photosynthesise under intensive sunlight, some reaching *P*_max_ at ~1000 µmol m^−2^ s^−1^ and dissipating extensive energy by non-photochemical quenching, NPQ (Proctor 2000). However, those species in open environments often experience water deficit. On the other hand, bryophytes from shady forest sites are adapted to live under as little as ~100–300 µmol m^−2^ s^−1^ but more rarely have to sustain drought. In this sense, *Sphagna* species typical for lawns and carpets of open bogs have a very specific position, as they grow under intensive light usually yet with no water deficit. However, *Sphagnum* species of open peatlands are reported to have lower photosynthetic capacity and maximum quantum yield than those that grow in the shade (Hájek et al. 2009).

Synergetic effects of solar UV with other co-varying environmental factors in bryophytes may be more pronounced (Sonesson et al. 2002; Rinnan et al. 2013) or even opposite (Björn et al. 1999) to those of UV alone. However, studies of such combined effects are scarce. Little is known about responses of different *Sphagnum* species to simultaneous changes in solar UV and temperature regime over a prolonged period of time (Sonesson et al. 2002; Aphalo 2003). UVB attenuation may have similar effects on peatmosses as increased summer temperatures, that include e.g. increasing length increment and simultaneously decreasing the bulk density of moss cover (Dorrepaal et al. 2003; Robson et al. 2003, 2004). To study the impact of UVB and temperature on peatmosses, we conducted an open field experiment on a flark fen in northern Finland. We aimed to study how changes in both UVB and temperature regime affect concentrations of chl *a* and *b*, and their ratio in three *Sphagnum* species: *S. balticum*, *S. jensenii* and *S. lindbergii*. Although varying in coverage, these were the most common peat moss species on the experimental site.

We hypothesized that under attenuated solar UVB and slightly elevated temperature chlorophyll production in *Sphagnum* mosses would increase. We suggested that UVB-attenuation plots might provide the most favourable conditions for plant growth, because of the release of apparent negative UVB effects and a parallel increase in temperature. Additionally, the polyester filters used for creating −UVB conditions partly transmit UVA, which may have positive effect on plants (Niemi et al. 2002b). Hence, we expected a higher chlorophyll concentration in the mosses growing in the UVB-attenuation plots.

## Methods

The UVB attenuation experiment took place in northern Finland on a remote oligotrophic tall-sedge *Sphagnum* flark fen, in Kaltiovuoma, Vuotso, about 200 km from Polar Circle [263 m a.s.l., 68˚10′N, 26˚42′E (75649:34878)] (Soppela et al. 2006). This open field experiment was a part of the ECOREIN project (The ecological and socioeconomical responses of global change on reindeer pastures). Therefore, the experimental site represented a typical reindeer summer pasture, where peat mosses dominated in the ground layer, most abundant being *S. lindbergii*, *S. jensenii*, S*. angustifolium*, and *S. balticum.* The field layer was characterized by sedges (*Eriophorum russeolum*, *E. angustifolium*, *Carex rotundata*, *C. rostrata*, *C. limosa*, *C. magellanica* subsp. *irrigua*, *Scheuchzeria palustris*), herbs (*Menyanthes trifoliata*, *Rubus chamaemorus*) and shrubs (*Andromeda polifolia*, *Betula nana*, *Vaccinium oxycoccus*, *V. microcarpum*). Chemical analysis of the upper substrate layer (0–30 cm) gave the following results: pH 4.2, carbon (total organic carbon) 41.5 %, nitrogen 0.64 %, carbon/nitrogen ratio 65, ammonium (NH_4_) 82.9 mg kg^−1^, nitrate (NO_3_) <0.2 mg kg^−1^ and ammonium-lactate extracted (i.e., plant-available) nutrients: phosphorous 45 mg kg^−1^, calcium 1.680 mg kg^−1^, magnesium 568 mg kg^−1^ and potassium 133 mg kg^−1^ (Soppela et al. 2006; Martz et al. 2009).

### Experimental design

**Fig. 1:**
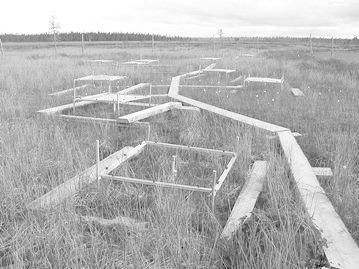
View of some of the plots and the walkway in the UVB attenuation experiment in Vuotso, northern Finland

In 2001, a site of about one hectare was fenced off on the area, to protect it from reindeer. In 2002, 30 randomly arranged plots were established on the site (Fig. 1). Each plot was surrounded by a wooden frame (100 × 100 cm). Three treatments were used in the experiment: (1) attenuated UVB (−UVB) plots, with frames covered by a clear polyester film, 0.125 mm thick (Polifoil, KTA Ltd., Helsinki, Finland), (2) control plots, covered with a clear solvent cast acetate film (Clarifoil, Derby, UK), and (3) ambient plots where frames were not covered. Each treatment was replicated ten times. The polyester transmitted less than 1 % of UV radiation below 315 nm, 30 % at 320 nm and about 70 % above 330 nm, while the cellulose acetate filter transmitted irradiance above 290 nm efficiently but less than 1 % of UV radiation below 290 nm (Soppela et al. 2006). However, due to diffuse radiation, actual UVB values reaching the mosses under the filters were higher. Under the polyester filters the radiation was on average 20 % and under cellulose acetate filters about 72 % of that in ambient plots (12.1 ± 0.3 uW/cm^2^ in –UVB, 42.1 ± 1.1 uW/cm^2^ in control, and 58.6 ± 3.1 uW/cm^2^ in ambient plots, mean ± SE).

**Fig. 2:**
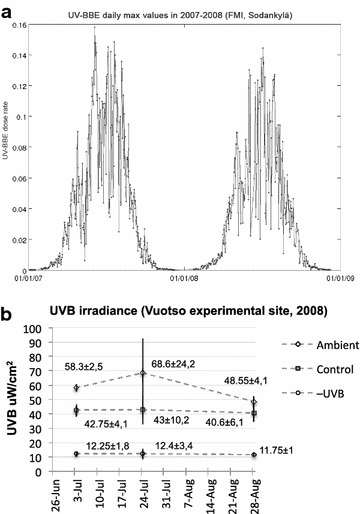
**a** Solar UV-BBE daily maxima in 2007–2008 (FMI, Sodankylä). **b** Solar UVB irradiance measured in Vuotso experimental site in 2008. The labels present mean data ±1 SD

**Fig. 3:**
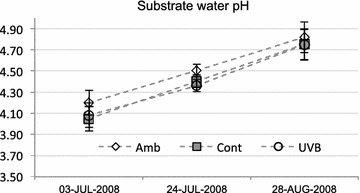
pH measurements in the field showed that substrate acidity did not significantly differ in different treatments. Values are estimated marginal means ± 90 % confidence intervals

Ambient UV irradiance was measured with a spectrophotometer Brewer MKII (SCI-TEC, Canada), installed on the roof of a building at the Arctic Research Centre of the Finnish Meteorological Institute (FMI-ARC) in Tähtelä, Sodankylä (67˚22′N, 26˚38′E), as described in Martz et al. (2007). The ambient biologically effective UVB (UV-BBE) irradiance was calculated according to Caldwell et al. (1980), and refers to UV-BBE calculated with the generalized plant action spectrum (Caldwell 1971) (Fig. 2a). We also performed UVB measurements in the field in 2008, while sampling, using a portable meter AIRAM UVM-8 (Kara Tekniikka Oy, Helsinki), equipped with a UVM-8B sensor (wavelength area: 270–370 nm, max sensitivity 310 nm) (Fig. 2b). Additionally, we measured the pH of the substrate water with a portable pH meter (Testo 206, Lenzkirch, Germany) (Fig. 3).

Each plot was equipped with a portable temperature datalogger (Tiny Talk II, −40 to 75 °C; OTLM Software, Orion Components, Ltd., Chichester, UK), attached to the central pole about 15 cm below the filter and 15 cm above the surface of the *Sphagnum* layer at the beginning of each growing season. Temperature was recorded every 1.5 h. Due to the film cover, the temperature in −UVB and control plots increased up to 1 °C, compared to the ambient plots. The average temperature for the period of record in 2008 (14th June–27th August) was 13.8 ± 0.09 °C in ambient, 14.8 ± 0.09 °C in control, and 14.5 ± 0.09 °C in –UVB plots (mean ± SE). Differences in temperature among the three treatments were statistically significant (F_2,26_ = 30.5, *p* < 0.001).

**Fig. 4:**
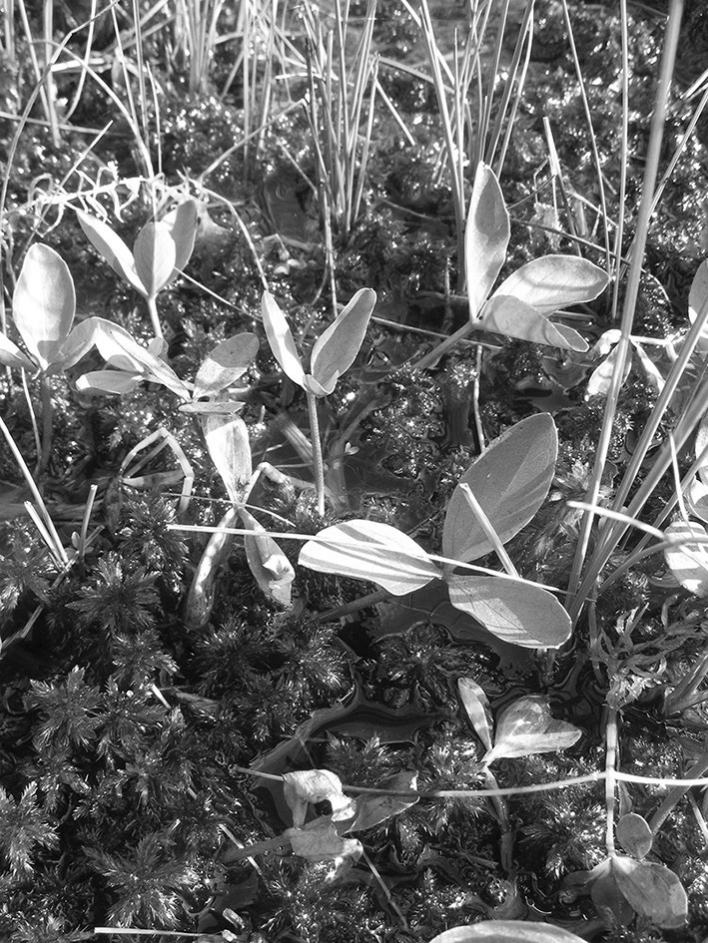
No additional water was given to the plots, because even in a very dry summer the water table in the fen remains stable under these cool and humid conditions. *Carex* sp., *Menyanthes trifoliata* and *Sphagna* seen in the picture seem to grow straight on water

Every year we re-installed the frames at the site, adjusting new plastic filters in May–June, as soon as weather conditions allowed access to the experimental site, but before the plants started to grow. During the growing season, the frames were gradually raised, to allow the tallest sedges to grow freely under the films. The filters were also slightly elevated in the center by a vertical pole so that the rainwater drained to the sides, keeping the filter dry. No additional water was given to the plots, because even in a very dry summer the water table in the fen remains stable (Fig. 4). The experimental periods were as follows: 6 June–5 September 2002 (91 days), 19 May–3 September 2003 (107 days), 17 June–6 September 2004 (81 days), 29 June–9 September 2005 (72 days), 31 May–18 September 2006 (110 days), 16 June–20 September 2007 (96 days), 14 June–28 August 2008 (74 days).

### Sampling and measurements

For chlorophyll analysis, we chose *S. lindbergii*, *S. jensenii*, and *S. balticum*, which make up, respectively 87, 5 and 3 % of the ground cover in the experimental plots (cover percentages calculated as a mean value from the 30 sample plots). We sampled *S. lindbergii* (*n* = 8–10 samples per treatment) in 2007 (12 July, 26 July, 23 August, 27 September) and 2008 (13 June, 3 July, 24 July, 28 August) to follow both seasonal and year-to-year changes in chlorophyll concentration. Each sample is a pooled sample of 10 capitula collected from each of the 30 experimental plots. When analysing year-to-year changes in chl concentration, differences in the sampling dates between the 2 years had to be taken into account. To analyse interspecies and seasonal variation, we also collected *S. balticum* and *S. jensenii* (*n* = 5–10 per treatment, due to smaller coverage of the species in the plots) in 2008 (3 July, 24 July, 28 August). The samples were immediately immersed in liquid nitrogen and transported to the laboratory of the Natural Resources Institute Finland (Luke) in Rovaniemi. The material was kept in a super freezer (−80 °C) prior to analysis. It was then lyophilized and ground. Exactly 15 mg (DW) of each sample were scaled, and used for chlorophyll extraction with pure methanol (1.5 ml per sample) in 24 h, at +4 °C, in the dark. The absorbance of the methanol extract was measured at 652.0, 665.2 and 750 nm with a spectrophotometer (Shimadzu-1700), and the concentration of chlorophyll *a* and *b*, and total chlorophyll (µg ml^−1^) was calculated following Porra et al. (1989).

### Statistical analysis

**Fig. 5:**
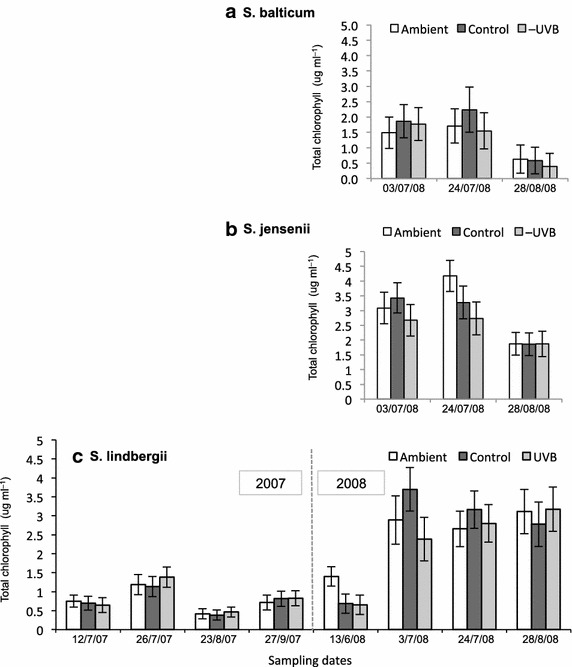
Seasonal variation of total chlorophyll content in **a** *Sphagnum balticum,* **b** *S. jensenii* and **c** *S. lindbergii* under ambient (*open bars*), control (*dark grey bars*) and −UVB treatment (*light grey bars*). For *S. lindbergii*, the data for 2007–2008 are shown. Values are estimated marginal means ± 90 % confidence intervals

**Fig. 6:**
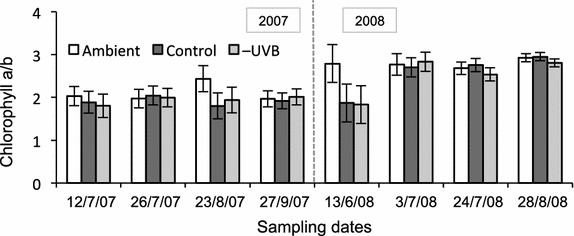
Interannual variation of chlorophyll *a/b* ratio in *S. lindbergii* under ambient (*open bars*), control (*dark grey bars*) and −UVB treatment (*light grey bars*). Values are estimated marginal means ± 90 % confidence intervals

The data were analysed by the means of linear mixed models in which the sampling date was taken as the repeated factor. In cases where the chlorophyll concentrations of all the three species were compared, both the species and the treatment were fixed factors. The species was not taken as a factor when analysing chlorophyll concentration in *S. lindbergii* alone, and substrate pH under different treatments. Due to the repetitive sampling from the same plots the data contained a correlation structure. The covariance structure for the repeated factor was heterogeneous first-order autoregressive (ARH1), which was selected on the basis of the lowest Akaike’s information criteria (AIC) value. For linear mixed models, Estimated Marginal Means (EMMEANS) and 90 % confidence intervals were computed, and are presented in Figs. 3, 5 and 6. Hence, those means with non-overlapping confidence intervals are statistically significantly different (at *p* < 0.05; Figs. 3, 5, 6). Correlations between environmental and chlorophyll concentration data were computed as Pearson’s correlation coefficients (Additional files 1, 2). Correlations between the chlorophyll content and substrate pH were computed as Spearman’s correlation coefficients. Statistical analyses of the data were performed using IBM SPSS Statistics 19.0.

## Results

### Seasonal and inter-annual variation

Prior to sampling, the three co-existing *Sphagnum* species grew under attenuated UVB and enhanced temperature conditions for six growing seasons (2002–2007). We detected significant species-specific seasonal variation in chlorophylls *a*, *b*, *a* + *b*, and chl *a/b* in these species during the experiment (2007–2008, Figs. 5, 6; Table 1). There was also significant inter-annual variation in *Sphagnum lindbergii* (Tables 1, 2; Figs. 5c, 6).

**Table 1:** Effect of UVB treatment, species, and sampling date on concentration of chlorophyll *a* and *b*, total chlorophyll (*a* + *b*) and *a/b* ratio

Source	Chl *a*		Chl *b*		Chl *a* + *b*		Chl *a*/*b*	
	*F*	*p* value	*F*	*p* value	*F*	*p* value	*F*	*p* value
Treatment	4.3	0.017	2.8	0.067	3.8	0.025	0.5	0.636
Species	52.1	<0.001	63.8	<0.001	56.4	<0.001	13.4	<0.001
Date	90.7	<0.001	79.9	0.000	90.8	<0.001	7.3	<0.001
Treatment × species	1.0	0.391	0.7	0.620	0.8	0.500	0.4	0.776
Treatment × date	3.4	0.003	2.3	0.037	3.2	0.005	1.7	0.124
Species × date	11.1	<0.001	7.2	<0.001	10.1	<0.001	8.3	<0.001
Treatment × species × date	2.0	0.050	1.6	0.127	1.8	0.088	4.7	<0.001

For each effect and their interactions *F* values and statistical significance of mixed linear model analysis are presented

**Table 2:** Effect of UVB treatment and sampling date on concentration of chlorophyll *a* and *b*, total chlorophyll (*a* + *b*) and *a/b* ratio in *Sphagnum lindbergii*

Source	Chl a		Chl b		Chl a + b		Chl a/b	
	*F*	*p* value	*F*	*p* value	*F*	*p* value	*F*	*p* value
Treatment	0.69	0.504	0.43	0.65	0.59	0.559	4.97	0.01
Date	66.7	<0.001	55.4	<0.001	64.5	<0.001	47.4	<0.001
Treatment × date	2.16	0.03	1.81	0.07	2.13	0.032	1.48	0.158

For both effects and their interaction *F* values and statistical significance of mixed linear model analysis are presented

In 2008, concentration of chlorophyll in the dominant *S. lindbergii* increased sharply from very low concentrations in mid-June to between two and six times that amount by July, then remained fairly stable for the rest of the season. In both *S. jensenii* and *S. balticum*, chlorophyll concentration decreased significantly towards the end of the summer in all the treatments (Fig. 5). *S. balticum* had significantly lower chlorophyll values than the other two species.

In *S. lindbergii*, chlorophyll concentration was noticeably lower in 2007 compared to 2008 (Fig. 5c). It also varied significantly during the growing season of 2007, being highest in July, decreasing at the end of summer and increasing again in September. The chl *a/b* ratio in this species was significantly lower in 2007 than in 2008 (Fig. 6).

### Treatment effects

Throughout the experiment, combined temperature and UVB manipulation had only minor effects on the chosen *Sphagnum* species in this habitat, and these effects were date-dependent (Tables 1, 2; treatment effect includes both UVB and temperature manipulation). A transient UV effect was detected on the 3rd July 2008 in *S. lindbergii* as a higher chl *a* + *b* concentration in the plants under control conditions, compared to those in the UVB-attenuated treatment (Fig. 5c).

A significant temperature effect was observed in *S. lindbergii* in 2008 (Figs. 5c, 6). By the end of the growing season in 2007 (27th September), plants under different treatments had approximately the same concentration of total chlorophyll and chl *a/b* ratio. However, when the next season started (13th June 2008), chlorophyll concentration and *a/b* ratio increased significantly in only the ambient plots, while in the plants under the filters these parameters remained at the level of the previous year. By contrast, later in the season of 2008, the chlorophyll concentration increased sharply (four–six fold) in *S. lindbergii* under the filters, while in ambient conditions less so (twofold). The chl *a/b* ratio in this species remained stable in ambient conditions for the whole growing season in 2008, while in the plots with increased temperature the ratio was significantly lower at the beginning of the season (Fig. 6). Additionally, the temperature effect was observed in *S. lindbergii* on 23rd August 2007, as a higher chl *a/b* in ambient compared to control plots.

## Discussion

**Fig. 7:**
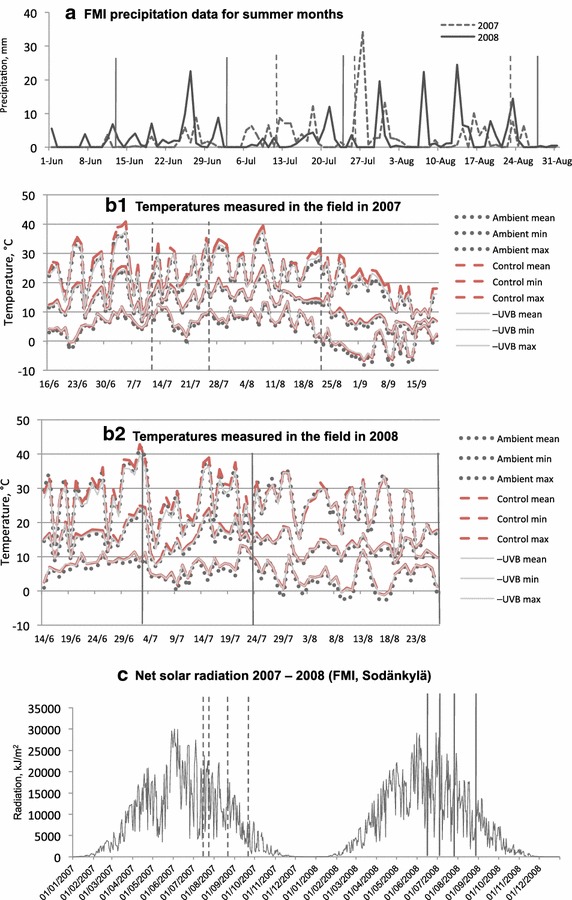
Environmental data: **a** Precipitation in June–August 2007–2008 (FMI, Sodankylä). **b** Temperatures, measured during the growing season in the experimental site in Vuotso. Mean, maximum and minimum values in different treatments are shown. **c** Daily sum of net radiation, normalized, kJ/m^2^ (FMI, Sodankylä). *Vertical lines* point the sampling dates (*dashed* for 2007, *solid* for 2008)

Seasonality in physiological characteristics is typical for bryophytes, together with wide variation in light responses between the species (Proctor 2000). Where light intensity varies substantially during the growing season (for example, in a deciduous forest—due to canopy closure), chlorophyll content and chl *a/b* ratio in mosses change noticeably in line with light availability. In open peatlands, by contrast, *Sphagnum* mosses experience relatively high irradiance, which remains stable for a prolonged period of time. This may explain why in *S. lindbergii*, both total chlorophyll concentration and chl *a/b* ratio were fairly stable in July–August 2008 (Figs. 5c, 6). This is in compliance with the results found by Mishler and Oliver (1991) in their study of bryophytes in open sunny habitats. Higher and more stable total chlorophyll concentration and higher chl *a/b* ratio in *S. lindbergii* in 2008 compared to 2007 could have been due to higher (253 mm in 2008 vs. 220 mm in 2007) and more evenly distributed precipitation during summer that year (FMI 2007–2008) (Fig. 7a).

An increase in total chl with decreasing light intensity and day length may be expected in autumn (Kershaw and Webber 1986; Niemi et al. 2002b), as we recorded from the samples of *S. lindbergii* in autumn 2007. However, in *S. balticum* and *S. jensenii*, the total chl content fell at the end of the growing season, as consistent with the findings of Lappalainen et al. (2008) and Gerdol (1996). These species with low coverage on the site have more specific substrate requirements. *S. balticum* is usually found on very poor ombrotrophic bogs with substrate pH < 4.5, while *S. jensenii* although growing at wider pH range (4.1–5.9), prefers more minerotrophic conditions of poor and intermediate fens (Wojtuń et al. 2013). The dominant *S. lindbergii* is comparatively flexible, successfully growing in a wide variety of trophic conditions, from ombro- to minerotrophic sites, with pH of the substrate ranging within ca. 4.0–5.8 (Gunnarsson et al. 2004; Laine et al. 2009; Wojtuń et al. 2013). In the latter species, the chlorophyll concentration did not decrease at the end of the growing season in 2008. The differences between the species are clearly seen when analysing correlation between chlorophyll content and pH of the substrate water. A significant negative correlation was found between the content of chl *a* and chl *b* and substrate pH in both *S. balticum* and *S. jensenii*, whereas in *S. lindbergii* chl content did not seem to be correlated with pH in none of the treatments (Table 3).

**Table 3:** Correlation between pH of the substrate and chlorophyll content in the three co-existing *Sphagnum* species in ambient and experimentally altered temperature and UVB conditions

pH in a treatment	*S. balticum*		*S. jensenii*		*S. lindbergii*	
	Chl *a*	Chl *b*	Chl *a*	Chl *b*	Chl *a*	Chl *b*
Ambient						
Spearman’s rho	−.506**	−.535**	−.522**	−.494**	.150	.055
*p* value	.008	.005	.004	.007	.446	.781
*N*	26	26	29	29	28	28
Control						
Spearman’s rho	−.464*	−.630**	−.607**	−.566**	−.189	−.308
*p* value	.030	.002	.000	.001	.327	.105
*N*	22	22	29	29	29	29
–UVB						
Spearman’s rho	−.449*	−.594**	−.404*	−.326	.167	.217
*p* value	.024	.002	.041	.105	.387	.259
*N*	25	25	26	26	29	29

* Correlation is significant at the 0.05 level (2-tailed)

** Correlation is significant at the 0.01 level (2-tailed)

Temperature and light intensity together affect photosynthetic rates in mosses (Proctor 2011), and thus may have potential to affect chlorophyll content in *Sphagna*; temperature effects on photosynthetic pigments in peat mosses depend on light intensity (Haraguchi and Yamada 2011). Certain *Sphagnum* species are adapted to have high photosynthetic rates at high temperatures and intensive light, or at low temperatures and low light levels. Low temperatures at high light intensity may have a negative effect on their photosynthesis (Haraguchi and Yamada 2011) and may have been one of the reasons for low chlorophyll content observed in *S. lindbergii* in mid June 2008. Low chlorophyll content at the beginning of the season might also be due to active growth, when shoots are growing fast, and chlorophyll biosynthesis rates do not match those of shoot growth (Kershaw and Webber 1986). Later in the season, intensive light combined with high ambient temperature may explain the increase in total chlorophyll in *S. lindbergii* (Fig. 7).

During this experiment, the species-specific seasonal changes in chlorophyll concentrations observed were more pronounced than the treatment effects (Table 4). The studied peat mosses, coexisting in the same habitat, showed quite uniform response to experimentally altered conditions, but differed in their responses to natural seasonal changes in light and temperature regime. Temperature and UVB manipulation we applied seemed to play a lesser role in plant acclimation than ecophysiological characteristics of a species, the unique combination of its niche requirements. In the given conditions, *S. lindbergii* seemed to be able to acclimate to a wider spectrum of environmental conditions, and to withstand environmental changes better than the more ombrotrophic *S. balticum* or more minerotrophic *S. jensenii* (Gignac 2011). It is possible that the ability to sustain the level of chlorophyll during the growing season is one of the features that helps *S. lindbergii* to keep its dominant position, compared to the other two *Sphagna,* in the given habitat.

**Table 4:** Correlations (Pearson correlation coefficients) between the total chlorophyll concentration (Chl) of the three studied species and temperature and UVB on the three different dates the UVB was measured and samples collected

Date	Chl in *S. balticum*		Chl in *S. jensenii*		Chl in *S. lindbergii*	
	Temperature	UVB	Temperature	UVB	Temperature	UVB
3.7.2008	.238	−.169	.193	.148	.282	.431*
24.7.2008	.076	−.240	−.349	.351	.220	−.129
28.8.2008	−.214	.352	−.055	.092	.005	−.036

The temperature is the daily mean of the preceding date. Statistically significant (*p* < 0.05) correlation coefficients marked with asterisk

Initially we hypothesized that *Sphagnum* mosses growing in the UVB-attenuation plots would have higher chlorophyll content as compared to the mosses in the ambient or control conditions. However, measurements revealed a higher chlorophyll concentration and chl *a/b* ratio in *S. lindbergii* sampled from ambient plots at the beginning of the season in 2008. This may be due to better overwintering abilities and higher photosynthetic activity in the plants in ambient conditions compared to those growing under increased temperatures (Gunnarsson et al. 2004). Higher total chlorophyll content in *S. lindbergii* in the control compared to −UVB plots on 3rd of July 2008 may also be explained by the higher UVA radiation doses under cellulose acetate filters.

At the end of the growing season in 2008, we detected no statistical differences between the treatments; instead, there were significant differences among the species in each treatment. This indicated that it was not the changes in temperature and UVB irradiance that affected the chlorophyll seasonality most, but rather species adaptability and their habitat requirements.

## Conclusions

A slight temperature increase and/or a significant attenuation of solar UVB radiation (to less than 1 % of the ambient) had no clear effect on the chlorophyll concentration or its seasonal variation in the *Sphagnum* species growing in an open fen. Altogether, the species studied seemed to be adapted to the changes in the environment they were exposed to. Seasonal variation of chlorophyll concentration in *Sphagnum* mosses proved to be species-specific and may be linked to environmental requirements of the species. *S. lindbergii*, dominant on the experimental site, showed good adaptive capabilities, since it was able to sustain high chlorophyll concentration throughout the season irrespective of the treatment applied.

## Additional files

Additional file 1: Linear correlation between temperature, UVB radiation and total chlorophyll content in* Sphagnum balticum*,* S. jensenii* and* S. lindbergii* (adjusted for date and species). * Correlation is significant at the 0.05 level (2-tailed). Additional file 2: Linear correlation between temperature, UVB radiation and total chlorophyll content in* Sphagnum balticum*, *S. jensenii* and *S. lindbergii* (adjusted for date, treatment and species). * Correlation is significant at the 0.05 level (2-tailed) ** Correlation is significant at the 0.01 level (2-tailed).
