# Supplementary material for: Seasonal and inter-annual variation in the chlorophyll content of three co-existing Sphagnum species exceeds the effect of solar UV reduction in a subarctic peatland
Source: Springerplus. 2015 Sep 4;4:478. doi: 10.1186/s40064-015-1253-7 (PMC4559556; doi:10.1186/s40064-015-1253-7)
Supplement: Additional file 1: — Linear correlation between temperature, UVB radiation and total chlorophyll content in Sphagnum balticum, S. jensenii and S. lindbergii (adjusted for date and species). * Correlation is significant at the 0.05 level (2-tailed). [file 40064_2015_1253_MOESM1_ESM.pdf]

**Date**	**Statistics**	**Chl *ab* in *S. balticum***		**Chl *ab* in *S. jensenii***		**Chl *ab* in *S. lindbergii***	
		temperature	*UVB*	*temperature*	*UVB*	*temperature*	*UVB*
3.7.2008	Pearson coef.	,238	-,169	,193	,148	,282	**,431****
	p-value	,232	,463	,335	,523	,154	**,045**
	N	27	21	27	21	27	22
24.7.2008	Pearson coef.	,076	-,240	-,349	,351	,220	-,129
	p-value	,743	,282	,074	,067	,227	,514
	N	21	22	27	28	27	28
28.8.2008	Pearson coef.	-,214	,352	-,055	,092	,005	-,036
	p-value	,339	,100	,784	,640	,981	,848
	N	22	23	27	28	29	30
