# Supplementary material for: Seasonal and inter-annual variation in the chlorophyll content of three co-existing Sphagnum species exceeds the effect of solar UV reduction in a subarctic peatland
Source: Springerplus. 2015 Sep 4;4:478. doi: 10.1186/s40064-015-1253-7 (PMC4559556; doi:10.1186/s40064-015-1253-7)
Supplement: Additional file 2: — Linear correlation between temperature, UVB radiation and total chlorophyll content in Sphagnum balticum, S. jensenii and S. lindbergii (adjusted for date, treatment and species). * Correlation is significant at the 0.05 level (2-tailed) ** Correlation is significant at the 0.01 level (2-tailed). [file 40064_2015_1253_MOESM2_ESM.pdf]

Date	Treatment	Statistics	Chl *ab* in *S. balticum*		Chl *ab* in *S. jensenii*		Chl *ab* in *S. lindbergii*	
			temperature	*UVB*	*temperature*	*UVB*	*temperature*	*UVB*
3.7.2008	Ambient	Pearson coef.	,139	,680	-,315	-1,000**	,419	-1,000**
		p-value	,722	,524	,447	-	,349	**-**
		N	9	3	8	2	7	2
	Control	Pearson coef.	,358	,150	,548	,269	,255	,072
		p-value	,345	,700	,101	,453	,477	,843
		N	9	9	10	10	100	10
	–UVB	Pearson coef.	,003	-,130	-,119	-,189	,091	,185
		p-value	,933	,738	,761	,626	,803	,609
		N	9	9	9	9	10	10
24.7.2008	Ambient	Pearson coef.	,357	**-,700***	,266	-,240	,136	-,300
		p-value	,386	**,036**	,489	,504	,727	,399
		N	8	9	9	10	9	10
	Control	Pearson coef.	,600	,506	-,387	-,134	,201	-,200
		p-value	,285	,384	,304	,731	,605	,606
		N	5	5	9	9	9	9
	–UVB	Pearson coef.	-,678	,542	,182	,325	,023	,041
		p-value	,065	,166	,639	,394	,953	,918
		N	8	8	9	9	9	9
28.8.2008	Ambient	Pearson coef.	-,301	-,113	,615	,211	,645	,356
		p-value	,562	,810	,078	,558	,061	,313
		N	6	7	9	10	9	10
	Control	Pearson coef.	,306	,640	-,004	,579	,459	-,005
		p-value	,461	,087	,991	,080	,182	,988
		N	8	8	10	10	10	10
	–UVB	Pearson coef.	,444	-,125	,278	-,072	-,038	-,106
		p-value	,270	,767	,504	,865	,917	,771
		N	8	8	8	8	10	10
